# Omeprazole-associated rhabdomyolysis

**DOI:** 10.1186/s13054-014-0462-8

**Published:** 2014-07-24

**Authors:** Kumiko Tanaka, Taka-Aki Nakada, Ryuzo Abe, Sakae Itoga, Fumio Nomura, Shigeto Oda

**Affiliations:** Department of Emergency and Critical Care Medicine, Chiba University Graduate School of Medicine, 1-8-1 Inohana, Chuo, Chiba 260-8677 Japan; Department of Molecular Diagnosis, Chiba University Graduate School of Medicine, 1-8-1 Inohana, Chuo, Chiba 260-8677 Japan

Proton pump inhibitors (PPIs) are commonly used in ICUs. Here, we report a severe case of rhabdomyolysis associated with omeprazole. A 20-year-old man, who previously had been healthy, visited a hospital with epigastric pain. An upper gastrointestinal endoscopy revealed a duodenal ulcer in an active stage. He was admitted to the hospital and received intravenous omeprazole (20 mg) twice a day. On day 14 of admission, he developed muscular pain, predominantly in the lower extremities, and had elevated serum creatinine phosphokinase (CPK) (28,314 IU/L; normal is less than 25 IU/L) (Figure 1). The patient was transferred to the hospital’s ICU on day 16, since the serum CPK (112,240 IU/L) and myoglobin (25,082 ng/mL; normal is less than 154 ng/mL) levels were extremely high. After potential causes of elevated CPK were considered, omeprazole-associated rhabdomyolysis seemed the most probable diagnosis. We discontinued intravenous omeprazole administration and started aggressive fluid repletion, continuous renal replacement therapy, and urine alkalization. The CPK and myoglobin levels successively decreased and reached within the normal range on day 31. The patient recovered completely and was discharged on day 38.

**Figure 1:**
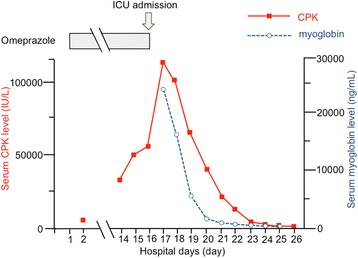
**Course of serum creatinine phosphokinase and myoglobin levels and intravenous administration of omeprazole.** CPK, creatinine phosphokinase.

No allergic symptom was detected in this case. The results of a drug-induced lymphocyte stimulation test for omeprazole were negative. Altered pharmacokinetics of omeprazole has been reported in patients with genetic variations in *CYP2C19*, which encodes a principal enzyme to metabolize omeprazole [1]; therefore, we performed DNA sequencing of the entire coding regions in *CYP2C19*. The analysis revealed no serious loss-of-function variations in the gene (intermediate metabolizer genotype) [1]. The plasma omeprazole level on day 15 was within normal range (380 ng/mL; normal is less than 400 ng/mL) [1]. Thus, the metabolism and plasma levels of omeprazole were not likely to be associated with rhabdomyolysis.

PPI-associated rhabdomyolysis is generally rare. This case had extremely high CPK/myoglobin levels compared with those reported earlier [2,3]. The mechanism of PPI-associated rhabdomyolysis has not yet been fully elucidated. Omeprazole is known to specifically bind to H^+^K^+^-ATPase at the gastric parietal cells. H^+^K^+^-ATPase is present in other tissues, including vascular smooth muscle cells [4]. Blocking H^+^K^+^-ATPase may induce artery vasoconstriction and ischemia, resulting in PPI-associated ocular damage [5], suggesting that a possible mechanism of PPI-associated rhabdomyolysis is via H^+^K^+^-ATPase in other tissues. Omeprazole activates gene expression of insulin-like growth factor-binding protein-1, a key mediator for muscle protein synthesis under stress [6], via the aryl hydrocarbon receptor [7]. The aryl hydrocarbon receptor pathway may involve PPI-associated rhabdomyolysis. We need to be aware of the possibilities, though rare, of rhabdomyolysis associated with omeprazole in the ICU.

## Abbreviations

CPK: Creatinine phosphokinase
PPI: Proton pump inhibitor
